# Evolution of an Epidermal Differentiation Complex (EDC) Gene Family in Birds

**DOI:** 10.3390/genes12050767

**Published:** 2021-05-18

**Authors:** Anthony Davis, Matthew J. Greenwold

**Affiliations:** 1 Department of Biological Sciences, University of South Carolina, Columbia, SC 29208, USA; davisableu@gmail.com; 2 Department of Biology, University of Texas at Tyler, Tyler, TX 75799, USA

**Keywords:** amniote, epidermis, genome, feathers, evolution

## Abstract

The transition of amniotes to a fully terrestrial lifestyle involved the adaptation of major molecular innovations to the epidermis, often in the form of epidermal appendages such as hair, scales and feathers. Feathers are diverse epidermal structures of birds, and their evolution has played a key role in the expansion of avian species to a wide range of lifestyles and habitats. As with other epidermal appendages, feather development is a complex process which involves many different genetic and protein elements. In mammals, many of the genetic elements involved in epidermal development are located at a specific genetic locus known as the epidermal differentiation complex (EDC). Studies have identified a homologous EDC locus in birds, which contains several genes expressed throughout epidermal and feather development. A family of avian EDC genes rich in aromatic amino acids that also contain MTF amino acid motifs (EDAAs/EDMTFs), that includes the previously reported histidine-rich or fast-protein (HRP/fp), an important marker in feather development, has expanded significantly in birds. Here, we characterize the EDAA gene family in birds and investigate the evolutionary history and possible functions of EDAA genes using phylogenetic and sequence analyses. We provide evidence that the EDAA gene family originated in an early archosaur ancestor, and has since expanded in birds, crocodiles and turtles, respectively. Furthermore, this study shows that the respective amino acid compositions of avian EDAAs are characteristic of structural functions associated with EDC genes and feather development. Finally, these results support the hypothesis that the genes of the EDC have evolved through tandem duplication and diversification, which has contributed to the evolution of the intricate avian epidermis and epidermal appendages.

## 1. Introduction

The adaptation of novel and complex appendages such as hair, scales and feathers were critical in the evolution of amniotes into a variety of terrestrial lifestyles [1,2,3]. The epidermal appendages of amniotes exhibit a wide range of physical properties that serve a variety of functions including but not limited to thermoregulation, camouflage and mating [4]. Generally, epidermal appendages form as the result of spatiotemporal interactions between cells of the epidermis and the underlying dermis, and the process involves several different genetic elements [5,6,7,8]. While the specific elements and processes involved in the development of epidermal appendages vary, evidence suggests that they all evolved from a single or small number of conserved ancestral gene(s) [9]. In amniotes such as mammals and reptiles, many of the genes encoding proteins involved in the mechanically resilient structure of epidermal appendages are found at a specific genetic locus known as the epidermal differentiation complex (EDC) [9,10,11,12,13,14].

One major reason for the evolutionary success of amniotic skin appendages is their unique and mechanically resilient physical properties [9,10,11,12,13,15]. To serve their various purposes, skin appendages tend to have increased tensile, flexural and yield strengths relative to the epidermis proper or internal organs, all of which have significant impacts on the physical characteristics exhibited by skin appendages [16]. These unique properties are largely the result of the evolution of novel and complex developmental processes that make use of structural proteins capable of covalently crosslinking with themselves and one another, often through transglutamination and disulfide bonding [1,17,18]. Studies have shown that differences in physical properties of different skin appendages can be correlated with differences in their respective amino acid contents. For example, Fujimoto et al. [19] found that the number of disulfide bonds formed by keratin-associated proteins enabled them to adhere to various structural proteins that they do not normally form associations with, indicating that the number and positions of conserved cysteine residues have a direct effect on the identity of the proteins involved in epidermal structure. These results suggest that differences between specific amino acid residues that are likely involved in protein crosslinking in structural genes could influence overall physical characteristics of the appendage in question [20,21].

The feathers of birds display a wide range of physical properties that have allowed birds to expand and survive in diverse environments across every continent including Antarctica [22]. Feathers were a critical adaptation in the evolution of avian flight, and the diversity observed across different species of birds’ feathers are a major reason for their ecological success. As with other epidermal appendages, many of the genes involved in the development and structure of feathers are located within the EDC locus and originated from a single or small number of ancestral genes [9]. The physical diversity observed across feathers is accompanied by the genetic diversity displayed by several differentially expressed avian EDC genes [9,23,24,25].

The avian EDC was first identified in the chicken (*Gallus gallus*) and was found to contain several genes that were characteristic of epidermal development and structure [9]. Several studies on the conservation of specific EDC genes identified in the chicken such as epidermal differentiation cysteine-rich protein (*EDCRP*), epidermal differentiation protein containing DPCC motifs (*EDDM*) and epidermal differentiation protein with an MTF motif rich in histidine (*EDMTFH*) have found that the EDC region as well as some specific genes are conserved across a broader range of avian species [8,20,21].

These studies found that while these genes were conserved across a broad range of avian species, there was significant sequence variation present. Moreover, studies on loricrins, a major component of the mammalian cornified envelope, in birds found that intragenic duplications of repetitive units have resulted in huge disparities in gene size, as well as a complex evolutionary history [22]. Additionally, this has led to a large diversity in sequence similarity across several EDC genes. 

Both intragenic and whole gene duplication have been shown to play major roles in the evolution of genetic diversity as well as in that of novel form and function [24]. The EDC locus has been found to have likely evolved through tandem gene duplication and diversification resulting in novel functions that contribute to the intricate avian epidermis [9]. Furthermore, studies have found that β-keratins, the primary protein component of the barbs and barbules of mature feathers, have diversified into several distinctly conserved subfamilies that have expanded outside of the EDC to other parts of the genome; however, they likely originated from ancestral genes within the EDC locus [25].

In contrast to *EDCRP*, *EDDM* and loricrins, which have evolved largely through intragenic duplications of repetitive units, other avian EDC genes represent members of conserved multigene families such as epidermal differentiation proteins containing cysteine histidine motifs (EDCHs) and epidermal differentiation proteins rich in aromatic amino acids and containing MTF motifs (EDAA/EDMTFs). These genes were originally identified and annotated by Strasser et al. [9] as only EDMTFs; however, the conserved “MTF motif” identified does not infer any specific functional motif, rather that the amino acid sequence of M-T-F was highly conserved in these genes. The EDAA/EDMTF genes are short sequences of less than 125 amino acids, which have been shown to be differentially expressed in developing feathers and scales of the chicken [8,9]. Specifically, *EDMTF4* and *EDMTFH* are highly expressed in the embryonic skin, feather and scale of the chicken while *EDMTF1* is highly expressed in the embryonic scale and beak of the chicken. *EDMTF4* and *EDMTFH* are lowly expressed in adult chicken feathers while *EDMTF4* is also lowly expressed in adult claw and embryonic beak [9]. Previous studies have found that EDAA/EDMTF genes are conserved across a diverse set of avian species as well as in crocodilians and turtles; however, little is known of their evolutionary history, function and conservation across a wider range of birds [11,13]. 

It is known that the evolution and expansion of the β-keratin multigene family, which originated within the EDC, was critical in the evolution of avian feathers [22,26,27,28,29,30]. Studies focusing on other conserved multigene families within the avian EDC would likely provide greater insight into the evolution of large, conserved groups of genes as well as their roles in the adaptation of novel structures such as feathers. In this study, we use phylogenetic and statistical analyses to more closely examine the evolution and conservation of the EDMTF genes in birds, as well as gain a better understanding of their possible functions in epidermal development. Furthermore, we provide a hypothesis that the evolution of novel structures such as feathers has largely been accompanied by the tandem duplication and diversification of EDC genes such as the EDAA/EDMTF gene family.

## 2. Materials and Methods

Avian EDAA/EDMTF genes were identified by BLAST+, specifically the *tblastn* command, which searches a nucleotide database using amino acid sequences as queries [31,32]. The amino acid sequences of chicken EDAA/EDMTF genes were used as the initial BLAST query; however, each identified sequence was added back to the query file and reciprocal rounds of BLAST searches were performed. In order to ensure no genes were missed, we used manual genomic screening methods, which entailed extracting entire genomic regions between two identified genes, and manually scanning the nucleotides for evidence of EDC genes not found by BLAST. No specific cutoff value or scores, such as e-value or BLAST score, were employed in these searches as they frequently resulted in little to no “hits”. The BLAST searches were used primarily to orient and identify the general EDC region in avian genomes, and the manual screening of those regions was the primary method for identifying genes. 

Suspected EDAA/EDMTF sequences were extracted as nucleotide FASTA files and translated to amino acids using the ExPasy Translate online analysis tool [33]. Translated amino acid sequences were characterized via multiple sequence alignment to chicken and other identified EDAA/EDMTF genes using the ClustalW online analysis tool [34]. To determine genomic orientation and the total number of EDAA/EDMTF genes in birds, manual screening was performed on genomic regions that had EDC BLAST hits. Table S1 details all the identified EMDTF genes, using the chicken as reference. Genes were considered complete if both N and C termini with start and stop codons were present as well as the minimal presence (<15%) of unknown nucleotides. Table S1 legend details the status and justification for all EDAA/EDMTF genes. Genes were considered incomplete if: 1—there were persistent unknown nucleotides within the coding sequence, 2—there was a frameshift present in the sequence that could not be resolved by switching reading frames, 3—no start codon was observed, 4—no stop codon was observed, 5—there was significant misalignment with reference sequences (i.e., no conserved elements of the gene in question were identified via alignment), and 6—there was a stop codon interrupting the ORF. The scores in Table S1 indicate the alignment score of each respective gene when aligned with that of the chicken (*Gallus gallus*).

**Figure 1:**
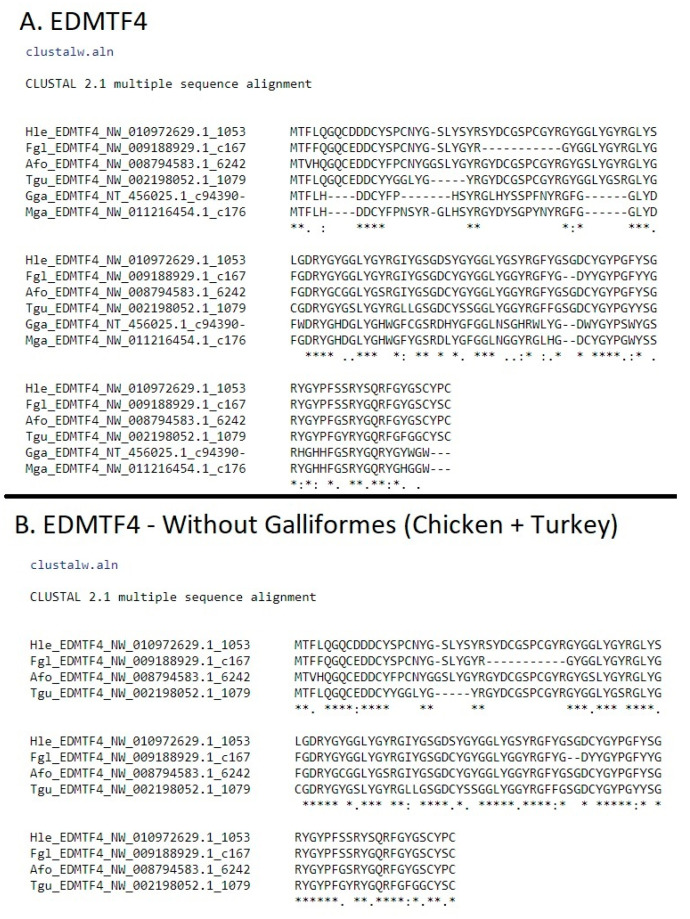
(**A**) Alignment of *EDMTF4* sequences from phylogenetically diverse group of birds (Afo: emperor penguin, Fgl: fulmar, Gga: chicken, Hle: bald eagle, Mga: turkey, Tgu: zebra finch). (**B**) Alignment of non-Galliforme *EDMTF4* genes. When Galliformes are removed from the alignment, there is much higher conservation of *EDMTF4*.

**Figure 2:**
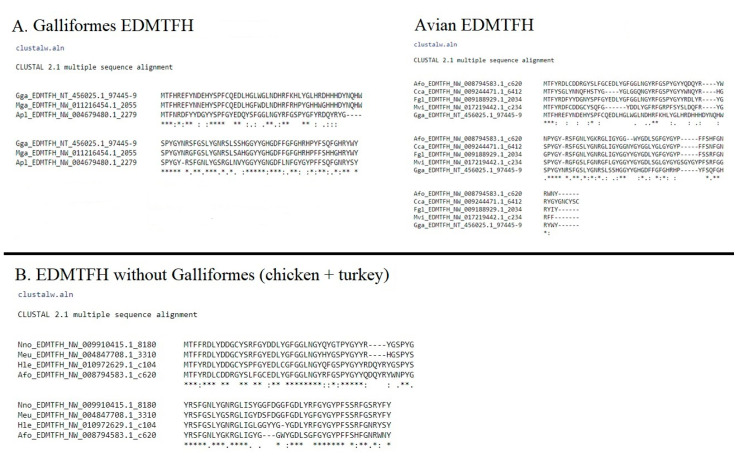
(**A**) Alignment of *EDMTFH* sequences from Galliformes (chicken and turkey) + Duck on left and additional species on right (Afo: emperor penguin, Apl: duck, Cca: will’s widow, Fgl: fulmar, Gga: chicken, Mvi: manakin, Mga: turkey). (**B**) Alignment of *EDMTFH* sequences minus the Galliformes. Indicates that, as with *EDMTF4*, there are differences in the amino acid content of *EDMTFH* genes; however, aromatic amino acid residues are conserved (Afo: emperor penguin, Hle: bald eagle, Meu: bee-eater, Nno: kea).

**Figure 3:**
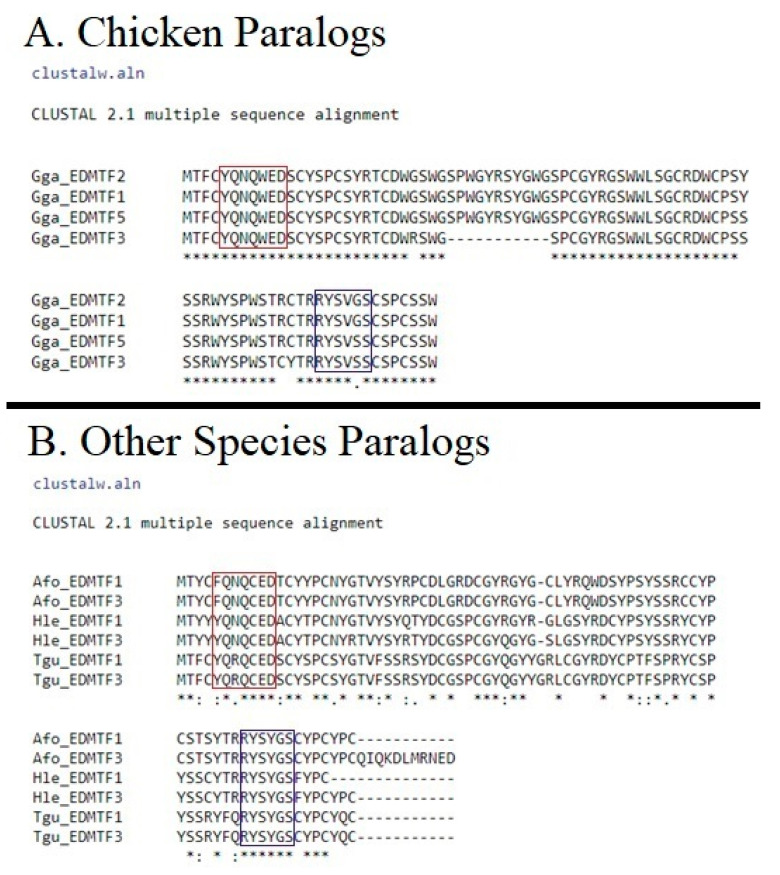
Alignment of *EDMTF1-3/5* paralogs. (**A**) The top alignment consists of the chicken paralogs *EDMTF1*, *EDMTF2*, *EDMTF3* and the newly identified *EDMTF5*. Alignment shows that with exception of small deletion in chicken *EDMTF3*, these genes represent duplicate genes. (**B**) Alignment of EDMTF paralogs from additional species (Afo: emperor penguin, Hle: bald eagle, and Tgu: zebra finch) demonstrate high lineage-specific conservation. Red and blue boxes indicate highly conserved sequences found across avian EDMTF genes.

Figure 1, Figure 2 and Figure 3 were aligned using the ClustalW online analysis tool [34] and figures were created and annotated using Microsoft Paint version 6.1. The architecture and orientation of avian EDAA/EDMTF loci were analyzed using chicken genes identified by Strasser et al. [9] as references. The identified genes were annotated based upon their position and genomic orientation corresponding to the chicken. Extra identified EDAA/EDMTF genes in addition to those in the chicken were also annotated based upon position and orientation. For example, the additional genes identified in the Cuckoo were annotated as *EDMTF1b* and *EDMTF1c* because they were located adjacent to EDMTF1 and in the same chromosomal orientation suggesting they are recent tandem duplications.

Phylogenetic analysis of avian EDAA/EDMTF genes was carried out using both Bayesian and maximum likelihood (ML) methods. Alignments of EDAA/EDMTF amino acid sequences were generated using ClustalW2 local alignment tool [34] and the alignments were edited using Bioedit 7.2 [35]. MEGA7 sequence analysis software [36] was used and identified PROTGAMMAJTT as the best fit substitution model based on Bayesian information criterion (BIC), Akaike information criterion corrected (AICc) and the substitution rate (BICJTT = 3849.826, AICcJTT = 2815.627). Bayesian analysis was carried out using Mrbayes-v3.2 [37,38] and was run for 10,000,000 generations and checked for convergence using the potential scale reduction factor method (PSRF) (TL:PSRF = 1.0; alpha: PSRF = 1.0). ML analysis was performed using RAxML-v8.2.10 [39] utilizing MRE-based bootstrapping until convergence was detected, followed by inferring the best tree produced out of 1000 generated ML trees, and finally mapping the MRE bootstrap values on the identified best tree. Sequences of EDAA/EDMTF genes from crocodilians and turtles identified by Holthaus et al. [11,13], respectively, were used as outgroups in both analyses. Avian sequences used in phylogenetic analyses were selected to represent a phylogenetically diverse group of bird species and lifestyles. All sequences used were considered complete and lacked unknown nucleotides. All sequences used in phylogenetic analysis are listed in Table S2. Trees were edited and viewed using FigTree-v1.4.3 [40].

Gene duplication dating of Common Cuckoo EDMTF genes was carried out using synonymous substitutions per site (K) estimates calculated in MEGA X [41] and mutation rate (r) estimates for the flycatcher (2.3 × 10^−9^ substitutions per site per year; [42]) and Galliformes (3.6 × 10^−9^ substitutions per site per year; [43]). The gene duplication time estimate was derived using the equation: r = K2/T [44], where T is the time estimate. The use of passerine and Galliforme mutation rate estimates were used as there are no available estimates of the cuckoo or other Columbaves. These estimates therefore have a range for each calculation. Amino acid analyses of avian EDAA/EDMTF genes were carried out using the ExPasy ProtParm online analysis tools [45]. The total number as well as overall percentage of each amino acid residue making up the ORFs of avian EDAA/EDMTF genes were calculated. The sequences used in amino acid analyses can be found in Table S2. To compensate for variation in the size of sequences across different species, we used the total percentage of each amino acid residue instead of the number. All sequences used were complete and contained no unknown nucleotides. Our overall amino acid composition analyses included 22 *EDMTFH* genes, 27 *EDMTF4* genes and 62 *EDMTF1*-*3*/*5* genes from 32 avian species.

Statistical analyses examining significant differences in amino acid contents of EDAA/EDMTF genes across different species, lifestyles and subfamilies was carried out using standard single factor analysis of variance (ANOVA) tests with the Microsoft Excel 2016 data analysis ToolPak. This ANOVA test was selected due to the small sample size available in the analyses. Principle component analysis (PCA) was carried out in R using the BiocLite-pcaMethods package (version 3.2) by BioConductor [46,47] using the singular value decomposition (SVD) method [48].

## 3. Results

### 3.1. The EDAA/EDMTF Gene Family Is Conserved in the Avian EDC

To better understand the evolution and function of the EDAA/EDMTF gene family, we screened the genomes of 48 phylogenetically diverse avian species for their presence using BLAST+ and manual genomic screening methods. We identified three major groups of EDAA/EDMTF genes across the birds investigated, the previously investigated *EDMTFH* (HRP) genes, *EDMTF4*s and finally *EDMTF1*-*3*/*5*+. These genes are annotated as described by Strasser et al. [9]. As expected, several genes identified were either partial or contained unknown sequence artifacts. Incomplete or partially identified genes were only used as evidence for the presence or absence of a specific genes and were excluded from amino acid and phylogenetic analyses. Each of the three major classes of EDAA/EDMTF, genes are characterized by distinct conserved sequence elements, genomic orientations and amino acid contents; however, there is considerable variation observed across different groups.

*EDMTF4* is generally characterized by highly conserved aspartic acid (D) residues in the N-terminal and central domains as well as the presence of several conserved tyrosine (Y) and glycine (G) throughout the gene (Figure 1a). While *EDMTF4* is conserved across all birds investigated, we found that *EDMTF4* of the chicken and turkey contain several conserved histidine residues are not present in other species, resulting in much greater conservation in *EDMTF4* sequence when the chicken and turkey are excluded from the analysis (Figure 1b). The species selected were used because they contain complete copies of EDAA genes and represent a diverse sampling of the entire bird phylogeny [30]. We found evidence for *EDMTF4* in all 48 species investigated; however, we identified partial or incomplete copies in nine species (Table S1). The table shows the presence of EDAA/EDMTF genes across birds investigated, their alignment scores relative to the corresponding gene in the chicken, as well as a descriptor if there was a problem or the gene was only partially found.

A previous study identified that the sequence of *EDMTFH* matches that of the previously reported histidine-rich protein (HRP), and it was conserved across a wide range of avian species [6]. Our results confirm the presence of *EDMTFH* in all species investigated by Alibardi et al. [6]; however, we did not identify any *EDMTFH* genes in passerine birds except for the golden-collared manakin (*Manacus vitellinus*). Evidence of *EDMTFH* was found in all the remaining 41 species, with three of those being partial or incomplete (Table S1). As reported by Alibardi et al. [6], only *EDMTFH* of the chicken and turkey was rich in histidine resulting in sequence variation; however, all *EDMTFH* genes identified contain the highly conserved sequence ‘-PYGYRsFGsLYGNRG-‘ within their central domains (Figure 2a). Outside of Galliformes, *EDMTFH* is highly conserved across all species investigated, except for the passerines (Figure 2b).

The final group of EDAA/EDMTF genes identified were *EDMTF1*-*3*/*5*. These genes are highly conserved across closely related species, and in many cases appear to represent species specific paralogs indicating a complex evolutionary history or possible concerted evolution. The most highly conserved elements of these genes across all species investigated were the presence of ‘-YQNQxED-‘ in the N-terminal region and ‘-RYSYGS-‘ in the C-terminal region; however, there is variation present across different species in the exact amino acid content and gene lengths, specifically in those of the Galliformes (Figure 3). All species except for the brown mesite (*Mesitornis uniclolor*) contained at least a single copy of these genes. Thirty-six of the 48 species contain the genes *EDMTF1* and *EDMTF3*, but no additional copies. Specifically, these species were missing the gene annotated as *EDMTF2* in the chicken. We did identify evidence of genes corresponding to the *EDMTF2* genomic position of the chicken in the golden-collared manakin (*Manacus vitellinus*), the Dalmatian Pelican (*Pelicanus crispus*), common cuckoo (*Cuculus canorus*) and Ostrich (*Struthio camelus*). Furthermore, we identified an additional copy of EDMTF, annotated as *EDMTF5* in the chicken (*Gallus gallus*) and two additional copies in the Common Cuckoo (*Cuculus canorus*) annotated as *EDMTF1b* and *EDMTF1c*. These genes were annotated based on their sequence elements and genomic orientation and are indicated in the table as “+ genes” (Table S1).

**Figure 4:**
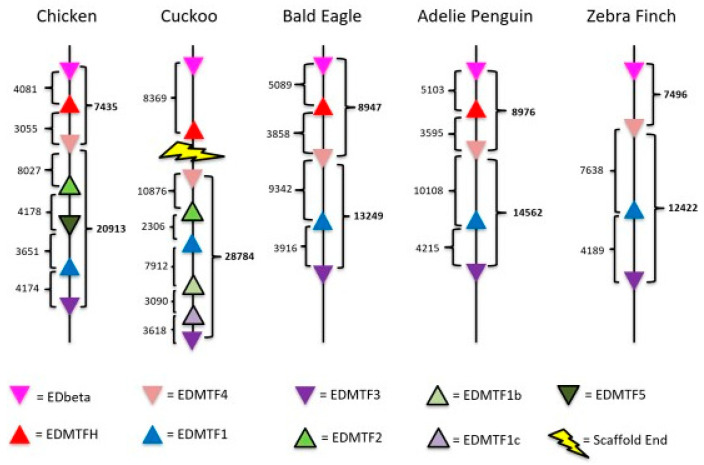
Overall conservation of genomic organization of EDAA/EDMTF gene family. The region of the EDC containing EDAA/EDMTF genes from five diverse bird species. The conserved β-keratin gene, *EDbeta*, is included for reference. Figure depicts variation in number of EDAA/EDMTF genes across different species as well as the variation in the overall size of this region. Brackets with numbers indicate the number of nucleotide residues between EDAA/EDMTF ORFs. The Cuckoo was the only species presented here where this entire region was not found on a single genomic scaffold.

The overall conservation of the EDAA/EDMTF gene family in five phylogenetically diverse birds is presented in Figure 4. Our results demonstrate that the EDAA/EDMTF gene family is conserved across birds, but with considerable variation. We found that there is variation in the overall size of this region across different avian EDC loci that corresponds to the number of genes found. For example, in the chicken and cuckoo, who contain additional copies of EDMTF genes, this region of the EDC contains 20,913 and 28,784 base pairs between *EDMTF4* and *EDMTF3*, respectively. In contrast, this EDC region of the bald eagle, Adelie penguin and zebra finch, which only possess *EDMTF4*/*1*/*3* are 13,249, 14,562, and 12,422 base pairs in length, respectively (Figure 4).

### 3.2. The EDAA/EDMTF Gene Family Originated in a Common Archosaur Ancestor

To investigate the evolutionary history of the EDAA/EDMTF gene family in birds and its role in the adaptation of complex appendages such as feathers and scales, we performed phylogenetic analyses using Bayesian and maximum-likelihood (ML) methods. Recent studies have identified homologous EDAA genes in the EDC loci of both crocodilians and turtles, and several of these genes were included in our analyses [9,11]. In total, we examined 149 EDAA/EDMTF genes including 108 avian genes from 28 different species, 22 from the painted turtle (*Chrysemys picta*) as well as 19 from two crocodilian species—the American alligator (*Alligator mississppiensis*, seven genes) and the saltwater crocodile (*Crocodylus porosus*, 12 genes) (Table S2).

**Figure 5:**
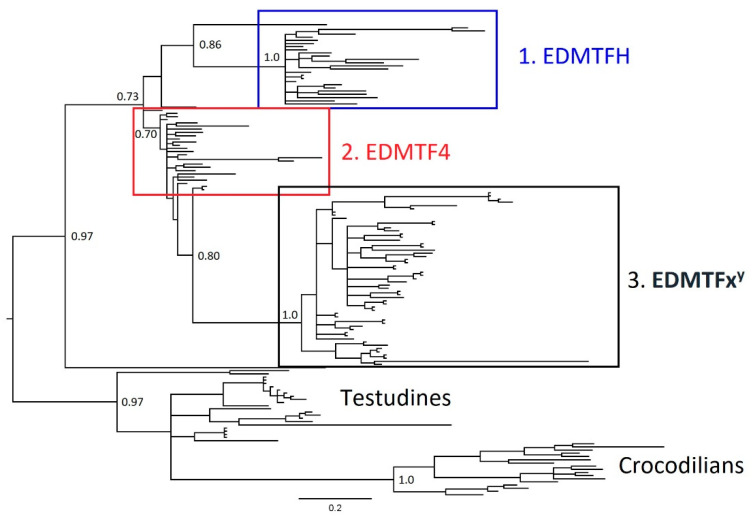
Bayesian phylogenetic analysis of EDAA/EDMTF gene family. Figure depicts Bayesian phylogenetic analysis of avian EDAA/EDMTF genes, using the EDAA genes of crocodilians and testudines as outgroups. The results demonstrate there are three conserved groups of avian EDAA/EDMTF genes. Group 1 contains avian *EDMFH* genes, group 2 contains *EDMTF4* genes and group 3 contains the remaining *EDMTF1*- *3*/*5* genes. Group 3 genes display a lineage-specific organization similar to that of *LOR3* and *LOR3B* genes in Davis et al. [22]. The turtle gene *cp_EDAA10* was located within the avian *EDMTFH* group and was the only non-avian species present in the three EDAA/EDMTF groups. Please also see Figure S1 for details on taxa labels and posterior probabilities for all nodes.

**Figure 6:**
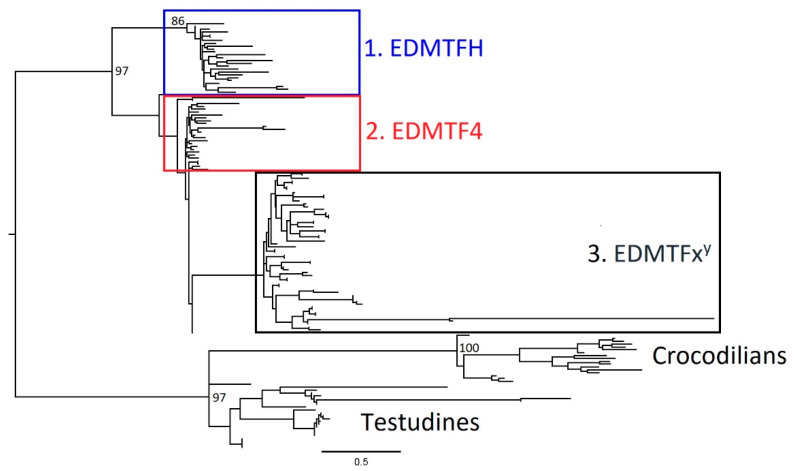
Maximum likelihood (ML) phylogenetic analysis of EDAA/EDMTF gene family. ML results display similar phylogenetic organization as Bayesian results confirming conservation of three distinct groups of avian EDAA/EDMTF genes. The turtle gene *cp_EDAA10* was in the avian *EDMTF4* group. This contrasted with the Bayesian analysis which placed this gene in the avian *EDMTFH* group. Please also see Figure S2 for details on taxa labels and posterior probabilities for all nodes.

In both ML and Bayesian analyses apart from *EDAA10* of the painted turtle, the EDAA genes of the crocodilians and turtles formed a large monophyletic clade with overall strong support and hence were selected as the outgroup. Our results confirmed the presence of three major groups of avian EDAA/EDMTF genes, *EDMTFH*, *EDMTF4* and then the additional *EDMTF1*-*3*/*5* genes (Figure 5 and Figure 6). In both analyses, *EDMTFH* formed a monophyletic clade with strong support values. *EDMTF4* and *EDMTF1*-*3*/*5* genes form a large clade, with *EDMTF4* representing a basal paraphyletic group and *EDMTF1*-*3*/*5* making up a monophyletic subclade; however, the support values associated with these groups are low. Interestingly, *EDAA10* of the painted turtle formed a monophyletic clade with *EDMTFH* in our Bayesian analysis, whereas in our ML analysis it was observed within the *EDMTF4* paraphyletic group, further highlighting the ambiguity associated with the low support values between the *EDMTFH* and *EDMTF4* clades.

In both ML and Bayesian analyses, the *EDMTF1*-*3*/*5* genes form a large monophyletic group (Figure 5 and Figure 6). Within this group, the genes display a lineage-specific distribution similar to that observed in avian loricrins [22]. The EDMTF genes of the Galliformes and Passerines form respective monophyletic groups within the major clade while all the remaining avian *EDMTF1*-*3*/*5* genes form a paraphyletic group. As diagrammed in Figure 4, the cuckoo has two additional EDMTF genes (*EDMTF1b* and *1c*) which together form a monophyletic clade with cuckoo *EDMTF1* and *2* genes (Figure S1 and S2). This alone suggests that the cuckoo has had multiple, recent gene duplications. We estimated the time of these duplication events as occurring in, at least, three separate time periods based upon nucleotide substitutions. We found that *EDMTF1b* and two genes are exact nucleotide matches and therefore represent a very recent duplication event. We also found that a duplication event occurred between ~2.5 and ~4.0 million years ago (MYA) and then one more between ~7.7 and ~16.0 MYA. 

This distribution largely agrees with the currently accepted species phylogeny of birds [30] and our own observations, which show that the sequences of EDMTF genes in Galliformes and Passeriformes contain unique amino acid contents relative to those of other species.

**Figure 7:**
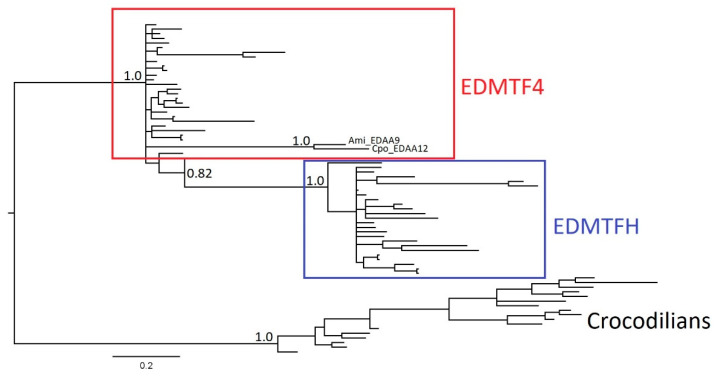
Bayesian phylogenetic analysis of *EDMTF4* and *EDMTFH* genes. Previously identified crocodilian EDAA genes were used as outgroups. In contrast with complete phylogenetic analyses, here avian *EDMTF4* is basal to *EDMTFH*. Interestingly, the crocodilian genes, *Ami_EDAA9* and *Cpo_EDAA12* were found in the avian *EDMTF4* group. Please also see Figure S3 for details on taxa labels and posterior probabilities for all nodes.

To better understand the origin of EDAA/EDMTF genes in birds as well as archosaurs in general, we further examined the evolutionary relationship of the avian *EDMTFH* and *EDMTF4* genes once again using the EDAA genes of crocodilians as the outgroup (Figure 7). Interestingly, two crocodilian genes, *EDAA9* of the American alligator and *EDAA12* of the saltwater crocodile, were present within the *EDMTF4* paraphyletic group. In the overall gene trees, these were the only crocodilian genes located outside of the crocodilian monophyletic group and instead were found within the turtle group (Figure S1, Figure S2, Figure 5 and Figure 6). As in our previous analysis, *EDMTFH* formed its own monophyletic group but was also part of a larger monophyletic clade with *EDMTF4*. This was in contrast with the previous analysis of all EDAA/EDMTF genes, where *EDMTFH* and *EDMTF4* formed a paralogous clade which excluded *EDMTF1*-*3*/*5* genes. The general support values for this tree were higher than those of the previous trees. All major branches contained values of 1.0 and the lowest support value observed was 0.5443 (*Ach_EDMTF4*) and was described for a terminal branch. All avian genes within the respective EDAA/EDMTF groups contained distinct groupings of the genes of Galliformes and Passeriformes, respectively, and this is largely in agreeance with the current avian species phylogeny proposed by Jarvis et al. [30].

### 3.3. EDAA/EDMTF Genes Contain Amino Acid Contents Indicative of Epidermal Development Structure

Previous studies have demonstrated that the avian EDAA/EDMTF genes are differentially expressed in developing chicken epidermal tissues [9]. It is also known that the amino acid contents of several other avian EDC genes vary significantly across different species [22]. This indicates that the amino acid composition of genes may correlate with their general function. To gain a better understanding of their possible function or functions in epidermal development of avian appendages, we analyzed the respective amino acid contents of the EDAA/EDMTF and performed statistical analyses including principal component analyses (PCA). Similar to our previous study examining avian loricrins [22], we report amino acid content as a percentage of specific residues instead of the exact number due to the variation in overall size of the coding sequences of EDAA/EDMTF genes across different species. In order to ensure accuracy in our analyses, only complete genes containing no unknown residues (XXXs) were analyzed here.

We analyzed the three main groups identified by phylogenetic analyses (Figure 5 and Figure 6) and found that all avian EDAA/EDMTF genes are rich in amino acid residues associated with epidermal structure and development processes [6,9,20,21]. The most abundant amino acid residues across all three groups were tyrosine (Y), glycine (G), serine (S) and cysteine (C) (Table S3). *EDMTFH* and *EDMTF4* contained similar amino acid contents, with tyrosine and glycine making up 41.82% (Y = 20.37%, σ = 3.44; G = 21.45%, σ = 3.32) and 49.12% (Y = 21.76%, σ = 2.36; G=27.36%, σ=2.22) of each respective gene. The main difference between the amino acid contents of *EDMTFH* and *EDMTF4* was the presence of increased cysteine in *EDMTF4* (*EDMTF4*:7.05%, σ=1.71; *EDMTFH*: 1.59%, σ = 0.92). Both genes contained similar average serine contents (*EDMTF4* = 8.93%, σ = 2.08; *EDMTFH*=8.47%, σ = 2.16). *EDMTF1*-*3*/*5* also was found to contain a very high tyrosine content, confirming that all genes were indeed rich in aromatic amino acids (Y = 22.19%, σ = 4.3). In contrast to *EDMTFH* and *EDMTF4*, *EDMTF1*-*3*/*5* was found to contain less glycine (G = 7.5%, σ = 2.19) as well as higher amounts of serine (S = 15.48, σ = 3.55) and cysteine (C = 15.17%, σ = 2.67; Table S3).

Alibardi et al. [6] found that the amino acid content of the *EDMTFH* gene was significantly different in the Galliformes (chicken and turkey) than in any other species.

Specifically, Galliforme *EDMTFH* are rich in histidine, whereas other avian *EDMTFH* genes contained little or no histidine. However, all *EDMTFH* genes were rich in aromatic amino acids. We found that a similar difference is observed in the *EDMTF4* amino acid composition of Galliformes relative to other avian species. Specifically, we observed significant differences in amino acid contents of cysteine (C; Galliformes C = 1.95%, σ = 0.071, n = 2; other C = 7.456%, σ = 0.904, n = 25; F25,2 = 71.512, *p* < 0.001), histidine (H; Galliformes H = 8.75%, σ = 1.77, n = 2; other H = 0.172%, σ = 0.43, n = 25; F25,2 = 450.8799, p < 0.001) and glycine (G; Galliformes G = 23.25%, σ = 3.182, n = 2; other G = 27.77%, σ = 1.84, n = 25; F25,2 = 9.89, *p* < 0.005).

**Figure 8:**
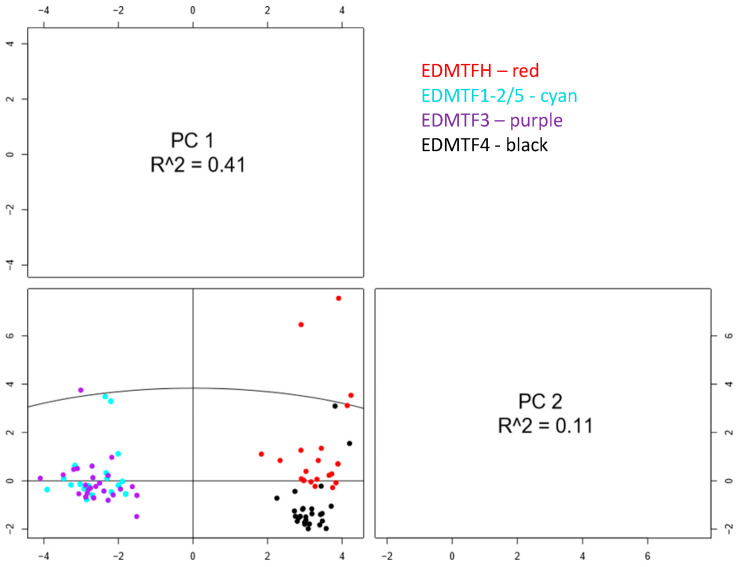
Principle component analysis (PCA) of avian EDAA/EDMTF gene amino acid contents. Results demonstrate that the amino acid contents of *EDMTFH* and *EDMTF4* are significantly distinct from those of *EDMTF1*-*3*/*5*. Additionally, *EDMTF4* and *EDMTFH* have conserved amino acid differences, though not as significant as compared with *EDMTF1*-*3*/*5*. Outliers likely represent the sequences of Galliformes, which have unique amino acid compositions but are still rich in aromatic amino acids.

**Figure 9:**
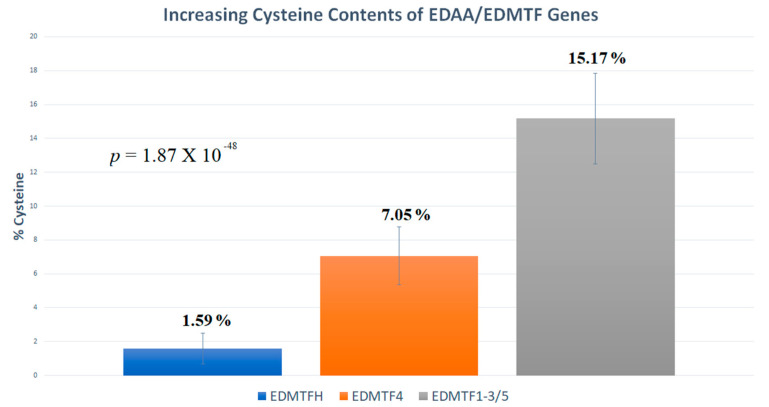
Significantly different cysteine content of avian EDAA/EDMTF genes. Bars indicate the percentage of the total coding sequence which is made up of cysteine residues across avian EDAA/EDMTF genes. The cysteine content of *EDMTF1*-*3*/*5* is much higher than other genes. ANOVA *p* = 1.87 × 10^−48^.

In order to further investigate the differences in evolutionary history identified by our phylogenetic analyses, we also performed a principal component analysis to further examine the differences observed between the amino acid compositions of avian EDAA/EDMTF genes. In this analysis, we also included the respective lengths of each gene as variables along with the amino acid residue percentages. The resulting PCA was graphed using two principal components which together described 52% of the total variation observed; however, PC1 was considerably more significant than PC2 (R2 PC1 = 0.41, PC2 = 0.11) (Figure 8). Our results confirmed that the three major groups of avian EDAA/EDMTF genes contained unique amino acid compositions. While a slight difference in amino acid contents would be expected given previous data, this method confirmed that this difference is significant. Furthermore, PCA analyses demonstrated that the amino acid contents of *EDMTF1*-*3*/*5* genes are significantly different from those of *EDMTFH* and *EDMTF4*, who possess similar amino acid contents. We observed 10 data points across all genes which displayed significant variation and could be considered to deviate from their respective groups (Figure 8). All but two of these 10 data points can be attributed to the significant diversity observed in the EDAA/EDMTF genes of Galliformes. We did not identify any significant groupings of respective genes based on avian lifestyles [25]; however, due to the limited number of complete genes identified from aquatic and predatory birds, more data is needed to further examine the possibility of a correlation between amino acid contents and lifestyle. We observed significant differences in amino acid contents across the different groups of genes. Specifically, there was a large difference in cysteine content, with *EDMTFH* containing significantly more cysteine than either *EDMTF4* or *EDMTF1*-*3* (Figure 9).

## 4. Discussion

In this article, we identified and characterized the EDAA/EDMTF gene family across a phylogenetically diverse set of avian species. Our results found that the EDAA/EDMTF gene family is conserved across birds and are rich in amino acid residues associated with epidermal structure and development [9]. These results provide new insights into the properties of specific EDC genes, as well as how the evolution and expansion of EDC genes has accompanied the adaptation of novel and complex skin appendages such as feathers.

Using genome screening, we identified EDAA/EDMTF homologs in every avian species investigated; however, there was variation in the number and identity of EDAA/EDMTF genes present. Previous studies identified five total EDAA/EDMTF genes in the chicken annotated as *EDMTFH*, *EDMTF4*, *EDMTF1*, *EDMTF2*, and *EDMTF3* [9]. We identified an additional duplicate of *EDMTF1*/*2*/*3* in the chicken that was not previously reported, which we annotated as *EDMTF5*. We found that the five EDAA/EDMTF genes identified in the chicken by Strasser et al. [9] and the additional *EDMTF5*, *EDMTFH*, *EDMTF4* and *EDMTF1*/*3* are conserved across birds; however, we found that no passerine birds possess an *EDMTFH* gene, except for the golden-collared manakin. Given our phylogenetic results and the overall conservation of *EDMTFH* in other avian species, the *EDMTFH* may have been lost in several lineages of passerines. Alternatively, and more likely, the *EDMTFH* gene is present within the genomes of passerine birds but due to problems with genomic library preparation and sequencing associated with EDC genes *EDMTFH* could not be identified in these genome assemblies [22,49].

Previous studies have provided evidence that *EDMTFH* is the earlier-reported histidine-rich protein (HRP) which has been suggested to be an important marker in early feather development [1,6,50]. Given that passerine birds are the most divergent and abundant order of birds, the failure to identify *EDMTFH* in several passerine species is interesting. As mentioned, Passerine birds make up 60% of extant birds and exhibit a vast amount of diversity across different lineages [51,52]. To date, no direct correlation between the absence of *EDMTFH* and any structural characteristics of passerine feathers has been identified; however, further studies investigating the timing and location of EDC gene expression could be of importance in answering this question. 

We found that both *EDMTFH* and *EDMTF4* have higher sequence similarity across species than *EDMTF1*-*3*/*5*, which displayed more lineage-specific sequence similarity, where genes in each respective species appeared to be duplicates. For example, we identified at least a single copy of *EDMTF1*-*3*/*5* in all species investigated except for the brown mesite, whereas *EDMTF2* was only identified in four species other than the chicken, indicating that the additional genes are the result of recent gene duplications and are not conserved across all birds. Furthermore, it is likely that *EDMTF2* of the cuckoo is not homologous with *EDMTF2* of the chicken, but instead is the result of a cuckoo-specific gene duplication event as it is an exact match to the cuckoo *EDMTF1b* gene. Furthermore, we found that another duplication event occurred in the cuckoo between ~2.5 and ~4.0 MYA, suggesting at least two species-specific duplication events and another older duplication event (~7.7–~16.0 MYA) occurring in the lineage leading to the common cuckoo. This is similar to the evolutionary history observed for avian loricrins, where although *LOR3* and *LOR3b* were conserved across birds, they appeared to be lineage-specific duplications [22]. The identification of the *EDMTF5* gene in the chicken, as well as the additional EDMTF genes in the cuckoo, ostrich, pelican and manakin, indicate that these genes are likely duplicating and expanding in many other avian species.

To better understand the evolutionary history and origin of the avian EDAA/EDMTF gene family, we examined sequences from phylogenetically diverse birds using both Bayesian and ML methods. Previous studies examining the EDC loci in crocodilians and turtles have identified homologous EDAA genes in syntenic locations within their EDCs and we included these genes as outgroups in our analysis. We found that there are three major groups of avian EDAA/EDMTF genes in birds (*EDMTFH*, *EDMTF4*, *EDMTF1*-*3*/*5*) and that they likely originated from a single or several ancestral archosaur EDC gene(s) similar to the evolutionary history of β-keratins described by Strasser et al. [9] and Greenwold et al. [25]. We hypothesize that the divergence of an ancestral archosaur gene resulted in *EDMTFH* in birds. Duplication and diversification of *EDMTFH* in birds resulted in *EDMTF4*, which was conserved across all species investigated. Further duplication and divergence of *EDMTF4* resulted in an ancestral form of the *EDMTF1*-*3*/*5* gene, which has continued to expand in some lineages such as the chicken and cuckoo. As mentioned previously, it is possible that at this point *EDMTFH* was lost in passerine birds except for the manakin, which may have retained this gene.

It is known that different amino acid composition of genes correlates with different functions, and therefore can also correlate with differential expression of related genes [53]. Strasser et al. [9] found that avian EDAA/EDMTF genes exhibited differential expression in developing epidermal tissues such as feathers, scales and skin. Furthermore, Strasser et al. [9] demonstrated that EDC genes across the chicken, anole lizard, and humans are enriched in residues such as glycine, serine, cysteine, proline, and glutamine. We analyzed the amino acid contents of the EDAA/EDMTF genes to look for significant variation in amino acid composition that could correlate with different functions. We found that the amino acid compositions of *EDMTFH* and *EDMTF4* were similar yet distinct, and significantly different from that of *EDMTF1*-*3*/*5* (Figure 8 and Figure 9). These results provide evidence that the differences in amino acid composition are significant enough to suggest differences in protein folding and composition. Differences in the folding of structural proteins such as loricrins have been shown to have an effect on physical and chemical properties of the proteins [15,22,53]. This is further supported by the results of Strasser et al. [9], which showed that the expression profiles for *EDMTFH* and *EDMTF4* were slightly different from one another and significantly different from *EDMTF1*, suggesting possible functional diversity during development. 

Our amino acid analyses identified the primary amino acid residues making up avian EDAA/EDMTF genes. Specifically, we found that the most prevalent amino acid residues across all EDAA/EDMTF genes are tyrosine, glycine, cysteine and serine. These residues are all known to be involved in epidermal development processes and mechanical structure. Tyrosine and glycine are both heavily involved in transglutamination, which has been demonstrated to play a major role in the mechanically resilient properties of the skin and appendages [54]. Cysteine residues are known to facilitate disulfide bonding, which has been shown to be important in feather and scale structure [17,54]. Finally, Serine has been found to be essential in epidermal development processes by facilitating serine protease activity, which is essential for the development of epidermal permeability and indispensable for postnatal survival [54,55].

Alibardi et al. [6] reported that *EDMTFH* of the chicken and the turkey contained a high amount of histidine, whereas *EDMTFH* of all other species contained little to no histidine; however, all were rich in aromatic amino acids. We identified a similar discrepancy in *EDMTF4*, which was histidine-rich in the chicken and turkey, but also contained much less cysteine relative to other species. Future studies comparing specific physical properties of feathers across different groups of birds such as Galliformes may identify the functional significance of amino acid differences in EDC genes. Further research is required to better understand the significance of the increased histidine contents of Galliformes’ *EDMTFH* and *EDMTF4*.

Our phylogenetic analysis of the EDAA/EDMTF gene family highlights a similar pattern of evolution with other avian EDC genes—evolution through tandem duplications and divergence. However, there are two major contrasting “types” of evolution observed. The first is what is observed primarily in the EDC genes *EDDM* and *EDCRP*, which are single genes conserved within the EDC of all birds, which have evolved primarily through tandem intragenic duplications [20,21]. The avian EDAA/EDMTF gene family, in contrast, has evolved largely through tandem gene duplication of entire genes. It is likely that the EDCH gene family described by Strasser et al. [9] also follows this method of evolution. Interestingly, we found that evolution of avian loricrins constitutes both of these models of evolution, where they have expanded into multiple conserved genes with differential expression, but they have also evolved through significant intragenic gene duplications, resulting in variation between species [9,22]. 

These results highlight the overall evolutionary history of the EDAA/EDMTF gene family and show that there are several similarities to the proposed evolutionary history of the β-keratin gene family. β-keratins are the primary protein component of mature barbs and barbules of feathers, and their genetic components have evolved into multiple conserved subfamilies [56,57]. Evidence suggests that all β-keratin subfamilies originated from a single or few β-keratin gene(s) located within the EDC of an ancestral archosaur and have since diversified to multiple genomic loci [20,25]. It is this diversification and expansion of differentially expressed β-keratin genes that is thought to have played a major role in the adaptation of birds to diverse lifestyles [25]. Our results show that the avian EDAA/EDMTF gene family also likely evolved from a single or small number of ancestral genes and has since expanded and diversified within the EDC locus into multiple conserved subgroups that are differentially expressed [9]. While there is no evidence that the EDAA/EDMTF genes have expanded outside the EDC, these results demonstrate that tandem duplication and divergence of genes has occurred frequently in the EDC. Given the importance of the EDAA/EDMTF genes in the epidermal development of birds, it is possible that these genes play a role in regulating developmental differences in birds. Further research is needed, however, to speculate about the specific function of the EDAA/EDMTF genes in feather structure and development, as well as the selective pressures driving their evolution.

## Data Availability

All sequences are available upon request as these were extracted from public genomes.

## Supplementary Materials

The following are available online at https://www.mdpi.com/article/10.3390/genes12050767/s1, Figure S1: high-resolution Bayesian phylogenetic analysis of the EDAA/EDMTF gene family, Figure S2: high-resolution maximum likelihood (ML) phylogenetic analysis of the EDAA/EDMTF gene family, Figure S3: Bayesian phylogenetic analysis of EDMTF4 and EDMTFH genes, Table S1: EDMTF genes presence/absence for 48 birds, Table S2: EDMTF gene annotation, Table S3: amino acid content for EDMTF genes.
