# Supplementary figures and images for: Evolution of an Epidermal Differentiation Complex (EDC) Gene Family in Birds
Source: Genes (Basel). 2021 May 18;12(5):767. doi: 10.3390/genes12050767 (PMC8157837; doi:10.3390/genes12050767)

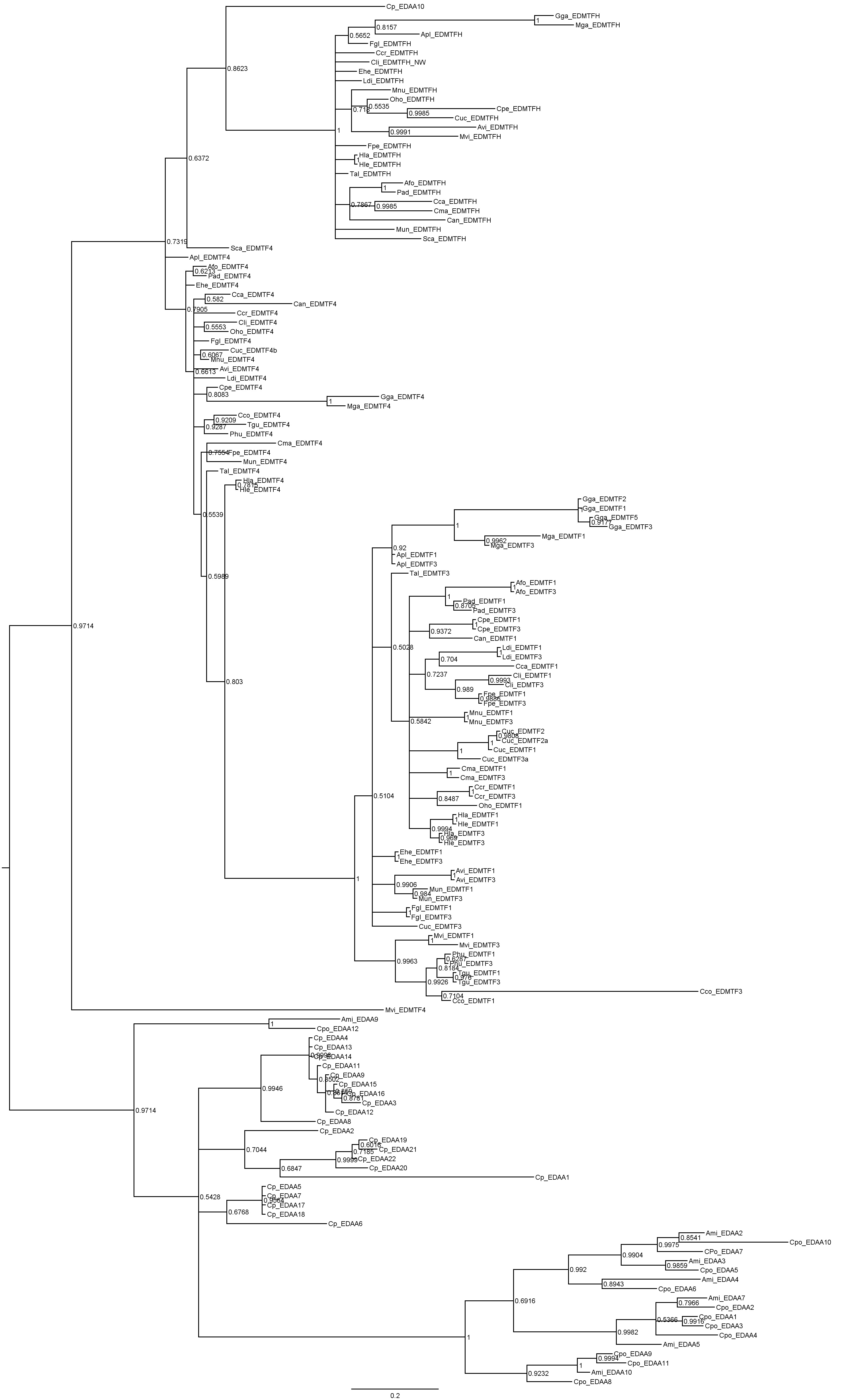

Supplement: Supplementary file 1 [file genes-12-00767-s001.zip › Figure_S1.jpg]

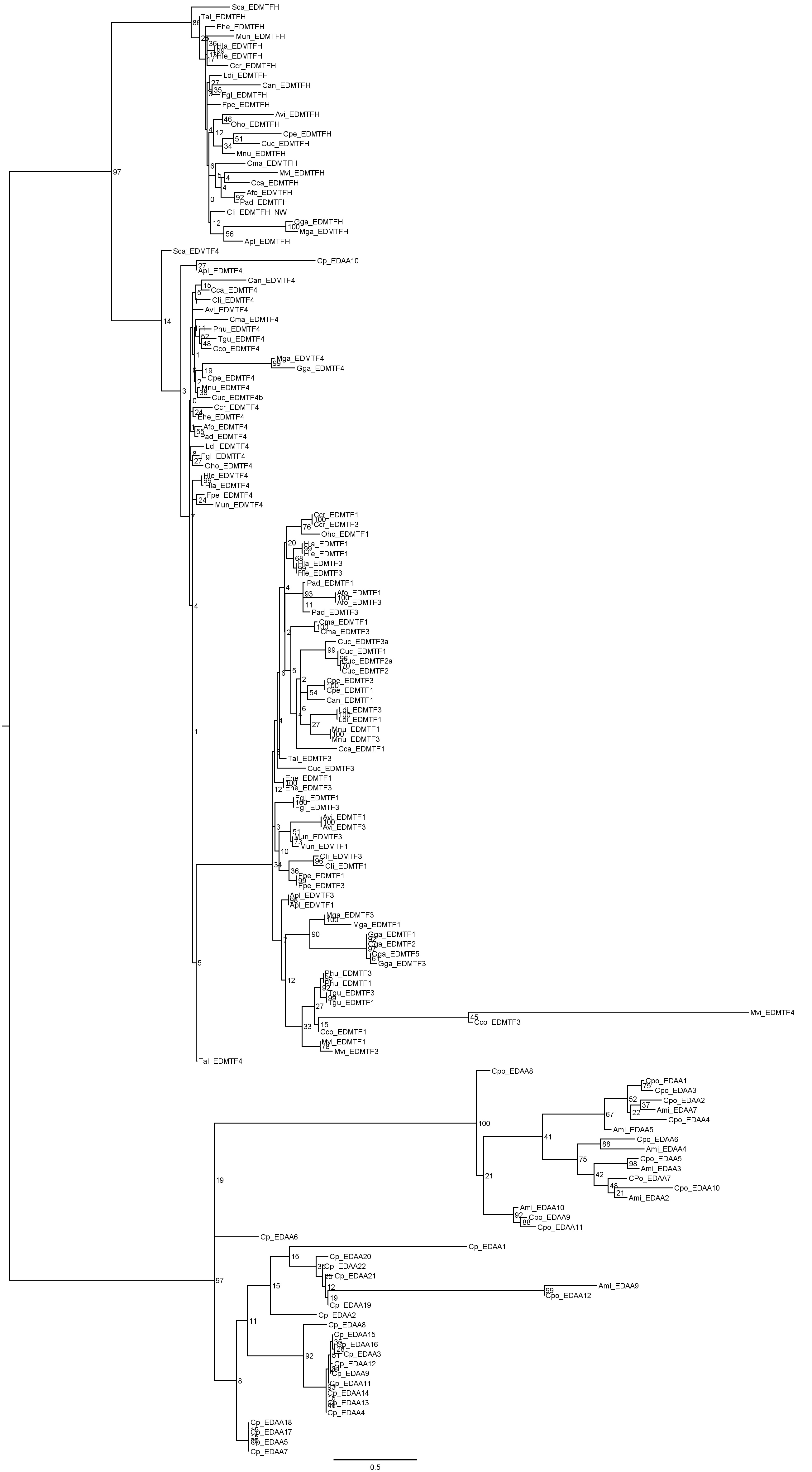

Supplement: Supplementary file 1 [file genes-12-00767-s001.zip › Figure_S2.jpg]

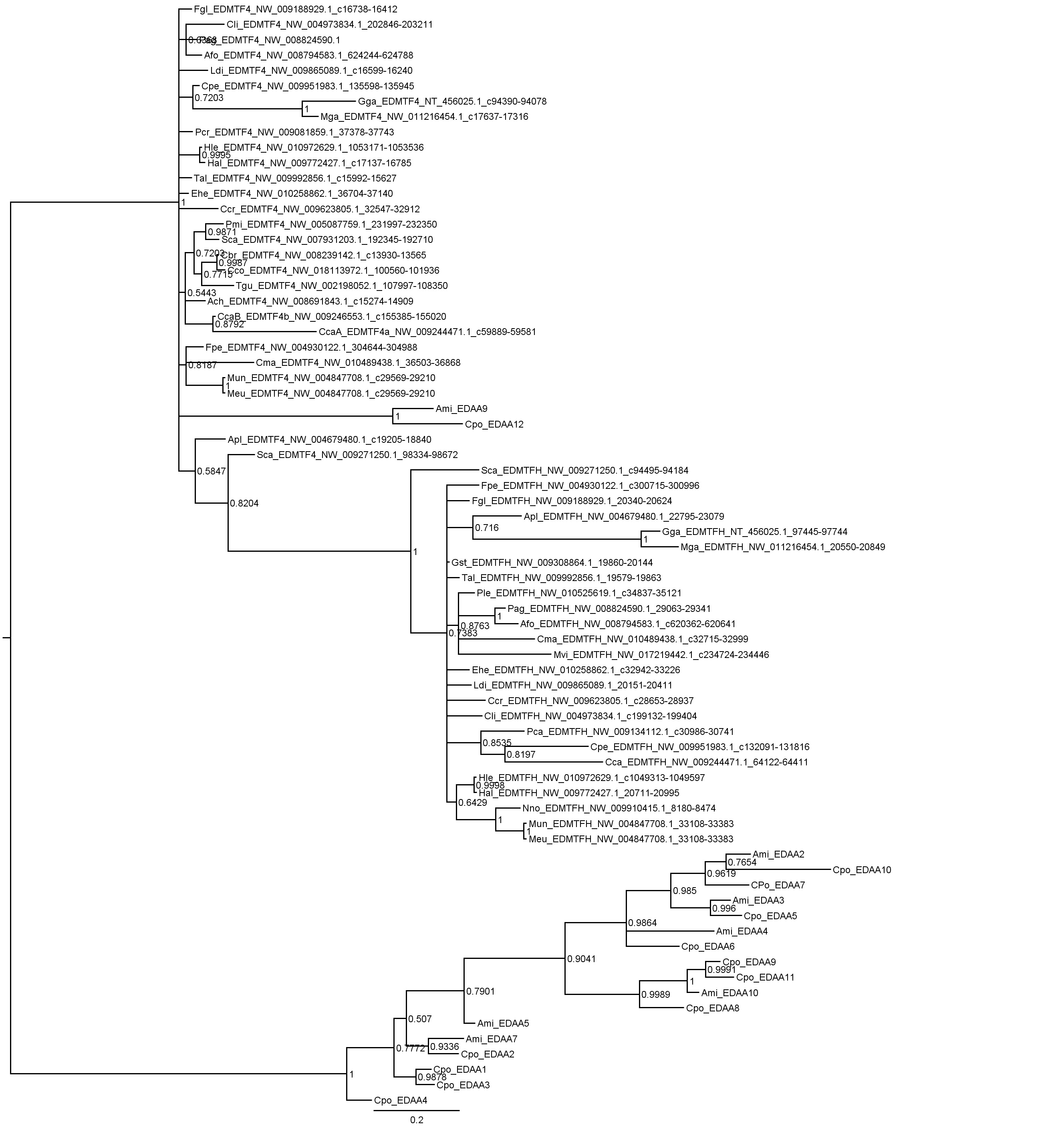

Supplement: Supplementary file 1 [file genes-12-00767-s001.zip › Figure_S3.jpg]
